# Studying genetic determinants of natural variation in human gene expression using Bayesian ANOVA

**DOI:** 10.1186/1753-6561-1-s1-s115

**Published:** 2007-12-18

**Authors:** Kevin C Cartier, Lara Miscimarra, Jean-Eudes Dazard, Yeunjoo Song, Sudha K Iyengar, J Sunil Rao

**Affiliations:** 1 Department of Epidemiology and Biostatistics, Case Western Reserve University, 10900 Euclid Avenue, Cleveland, Ohio 44106, USA

## Abstract

Standard genetic mapping techniques scan chromosomal segments for location of genetic linkage and association signals. The majority of these methods consider only correlations at single markers and/or phenotypes with explicit detailing of the genetic structure. These methods tend to be limited by their inability to consider the effect of large numbers of model variables jointly. In contrast, we propose a Bayesian analysis of variance (ANOVA) method to categorize individuals based on similarity of multidimensional profiles and attempt to analyze all variables simultaneously. Using Problem 1 of the Genetic Analysis Workshop 15 data set, we demonstrate the method's utility for joint analysis of gene expression levels and single-nucleotide polymorphism genotypes. We show that the method extracts similar information to that of previous genetic mapping analyses, and suggest extensions of the method for mining unique information not previously found.

## Background

The extent to which the natural variation in gene expression is heritable has recently been the subject of some intriguing studies [1-5] the most notable being a study by Morley et al. [6] in which expression phenotypes were analyzed as quantitative traits, yielding significant evidence for linkage between expression levels and single-nucleotide polymorphism (SNP) genotypes.

The Problem 1 data set for the Genetic Analysis Workshop 15 (GAW15) consists of 3554 lymphoblastoid gene expression values observed in a sample of 194 healthy individuals from 14 three-generation CEPH (Centre d'Etude du Polymorphisme Humain) Utah families. Additionally, the sample contains genotypes for 2882 autosomal and X-linked SNPs for each of these individuals. The challenge thereby presented is to measure and characterize the heritability of expression phenotypes.

In this paper we present our analysis of the GAW15 Problem 1 data set based on a variable-selection method tied to a Bayesian ANOVA model that has been shown to be particularly effective in separating signal from noise within expression data.

## Methods

### The multigroup ANOVA model

Let *Y*_*i*, *j *_be the gene expression value for the *j*^th ^gene on the *i*^th ^individual, where *j *= {1, 2,..., *M*} and *i *= {1, 2,..., *n*}. Further, let G_*i *_denote the group membership of the *i*^th ^individual, where G_*i *_={1, 2,..., *g*}, let **I**(·) denote an indicator function, and let *n*_*k *_= #{*i*: *G*_*i *_= *k*} denote the number of individuals belonging to group *k*, such that the total sample size is $n=\sum_{k=1}^{g}n_{k}$. Then the multigroup ANOVA model is given by

$$Y_{i,j}=\theta_{j}+\sum_{k=1}^{g-1}\beta_{k,j}I\{G_{i}=k\}+\varepsilon_{i,j},$$

where *θ*_*j *_is the baseline effect for the *j*^th ^gene, *β*_*k*, *j *_is the group-differential effect (comparing the *j*^th ^gene in group k against a baseline group) and *ε*_*i*, *j *_is the error term. The errors are assumed to be independently distributed with mean zero such that E[*ε*_*i*, *j*_] = 0 and Var[*ε*_*i*, *j*_] = *σ*_*j*_^2^. This means that every gene can have its own variance component. Aside from this, there are no distributional assumptions about the data.

This multigroup model can be restated as a linear model as described in Ishwaran and Rao [7] and the task of identifying differentially expressing genes amounts to finding non-zero group × gene interactions effects. A Bayesian variable selection technique described below can then be used to identify these parameters of interest. Despite relatively weak assumptions about the data generating mechanism, we will nevertheless require that a common variance model holds for the variable selection process, and it is therefore necessary to transform the data to stabilize the variances across genes. This is done using a weighted regression technique also described in Ishwaran and Rao [7], which has the advantage of avoiding problems associated with more typical global variance-stabilizing transformations (log-transformations, for example), but at the same time does not change the signal-to-noise ratio of the data for any given gene. Let *Y*_*i*, *j*_^+ ^denote data produced by this transformation, and select a baseline group *g*. Then the data are further centered and scaled by

$$\begin{array}{cc} Y_{i,j}^{*}=\sqrt{\frac{N}{{\hat{\sigma }}_{N}^{2}}}(Y_{i,j}^{+}-{\bar{Y}}_{g,j}^{+}), & G_{i}\neq g, \end{array}$$

where *N *= (*n *- *n*_*g*_)*M *is the total sample size and ${\hat{\sigma }}_{N}^{2}$ is an unbiased estimator for the common variance, ${\hat{\sigma }}_{0}^{2}$.

### Spike and slab hierarchical model for Bayesian ANOVA of microarrays (BAM)

Following some additional pre-processing steps [7] to generate an orthogonal linear model design, Ishwaran and Rao's *spike *and *slab *hierarchical model [7] is given as:

$$\begin{array}{c} \begin{array}{cc} (Y_{j}^{*}|\beta_{j},\sigma^{2})\overset{ind}{\sim }N(X_{j}\beta_{j},N\sigma^{2}I), & j=1,2,...,M \end{array}\\ (\beta_{j}|\gamma )\sim N(0,\Gamma_{j}),\\ \gamma \sim \pi (d\gamma ),\\ \sigma \sim \mu (d\sigma^{2}), \end{array}$$

where Γ_*j *_is the diagonal matrix with diagonal entries obtained from ***γ***_*j *_= (*γ*_1, *j*_, *γ*_2, *j*_,..., *γ*_*g*-1, *j*_)^*t*^, and ***γ ***= (*γ*_1_^*t*^, *γ*_2_^*t*^,..., *γ*_*M*_^*t*^)^*t *^is a *M*(*g *- 1) -dimensional hypervariance vector. Each design matrix ***X***_*j *_is chosen for orthogonality such that ***X***_*j*_^*t*^***X***_*j *_= *N****I***. The prior distribution for the coefficient variances, *π *(*dγ*), is a continuous bimodal prior in which one spike component specifies small values, thus favoring small values of *β*_*j*_, and a right continuous tail specifies preferences for large non-zero values of *β*_*j*_.

Ishwaran and Rao [8] derive a blocked Gibbs sampling algorithm for posterior inference and show that the posterior mean for the *β *values is the optimal summary measure to use. Hard thresholding is done using a data-adaptive rule based on looking for coalescence of the posterior variance at a value of 1.0 with corresponding large posterior mean values (see Ishwaran and Rao [7] for details). This spike and slab model was also shown to possess a selective shrinkage property where shrinkage towards 0 (from classical least square estimates) occurs only for those parameters corresponding to truly non-differentially expressing genes with probability tending to 1 as the sample size increases. The model described above is implemented in a software package called BAMarray [9], and is available for download at .

It should be noted that the posterior mean estimator has been shown to be a weighted average of generalized ridge regression estimators, hence justifying the distribution-free assumptions made in the original ANOVA model. So in effect, the hierarchical model is a tool to generate a Bayesian test statistic (referred to as a *Zcut *value [7]), and corresponding thresholding rule, but does not reflect our belief about the data generating mechanism.

### Robustness of spike and slab model

The model is robust to non-normality of the response variable, robust to *clumpy dependence *across genes [10] and allows unequal variances across genes. In addition, the spike and slab model was shown to be robust against correlation in gene expression measurements across individuals. Typically, when not accounted for, these correlations tend to produce underestimated variance estimates and can lead to detection of spurious effects. It was shown that the spike and slab model greatly mitigated this problem due to its selective shrinkage property, which shrinks only those coefficients corresponding to truly zero effects (see Theorem 1 and Remark 5 of Ishwaran and Rao [8]).

### Determining gene expression profiles

For our purposes, a gene *expression profile *is a multi-gene pattern of expression values by which a given individual may be assigned membership to one of a set of mutually exclusive groups or categories. Our strategy for determining expression profiles is to ask whether gene expression measurements for each individual can be classified as up, down or equi-regulated with respect to a "median" family expression profile. Interestingly, this can be handled as a special case of the multi-group model in what is termed a *no-baseline *analysis, and by accruing this information across genes we obtain a novel profile from which to assign individuals to expression profile clusters across families. This process allows us to define expression profiles using all of the genes present rather than trying to do things on a gene-by-gene basis. This also means that a parameter vector of length equal to the number of genes on the chip must be estimated for each individual. In order to conduct such an analysis, we extended the data set with pseudo samples obtained as follows: for each possible pairing of individuals within each pedigree, we calculate the pair-wise difference vector and add it to the existing data set. The effect of taking all possible pair-wise differences between family members is to increase the sample size while at the same time centering the expression profiles on a per-family basis. Each vector difference is added as a pseudo sample to the existing set of data, and for a pedigree of *K *individuals, the total number of such additions is (*K*)(*K*-1). Note also that with the generation of the pseudo samples and by use of the no-baseline variation of the spike and slab BAM model, we are implicitly allowing each individual to act as a "group".

#### Remark

The generation of pseudo samples and the inherent familial structure of the pedigrees means that the expression values will be correlated. But as previously indicated, the spike and slab BAM model is robust to these correlations and gives correct inferences.

After estimating Zcut vectors from the BAM no baseline model for each individual, we clustered those vectors across individuals using agglomerative hierarchical clustering, wherein the optimal number of clusters is determined by Tibshirani's *gap statistic *[11]. In order to determine which genes were most influential in the clustering (i.e., generation of expression profiles) we examined each gene via a one-way ANOVA F statistic using the assigned latent cluster labels as group indicators. Corresponding *p*-values were determined and then adjusted for multiplicity by using the Benjamini-Hochberg version of false detection rate (FDR) control [12].

### Relating the latent gene expression clusters to the SNP genotype information

Using the GAW15 data set, we sought to relate gene expression to SNP genotypes collected from pedigrees of healthy individuals. In essence, we were trying to determine the genetic determinants of the natural variation of gene expression in this data set. Given that we do not have explicit groupings of individuals into different phenotypic groups (e.g., case/control) our approach was to use the clustered expression profiles described above and relate these latent clusters to the SNP genotype information using the spike and slab multi-group methodology described above. Similar arguments about correlation between SNP measurements across individuals still hold but the correlations across individuals within the latent expression profile clusters are expected to be less strong than the expression correlations within same pedigree. We can apply the spike and slab BAM model to SNP data because the model does not make distributional assumptions about the data generating mechanism, and employs a limiting distribution result for its thresholding rule.

## Results

We began by generating the additional pseudo samples as described above, and were thus able to increase the analyzable sample size from the original 194 records to 2496. On average, each individual from the original data set was mapped to a unique set of 182 expression profile records. The no-baseline analysis on these data revealed that, on average, 30% of each individual's genes make a significant contribution to profile differentiation. At the same time, none of the genes examined could be immediately eliminated from further consideration. In other words, there were no genes with Zcut values equal to zero for all individuals in the sample. The sample proportion of non-zero Zcut values, on a per-gene basis, ranged from 18% to 88%, indicating that some genes are likely to appear in all expression profiles, while others are more discriminatory.

**Figure 1:**
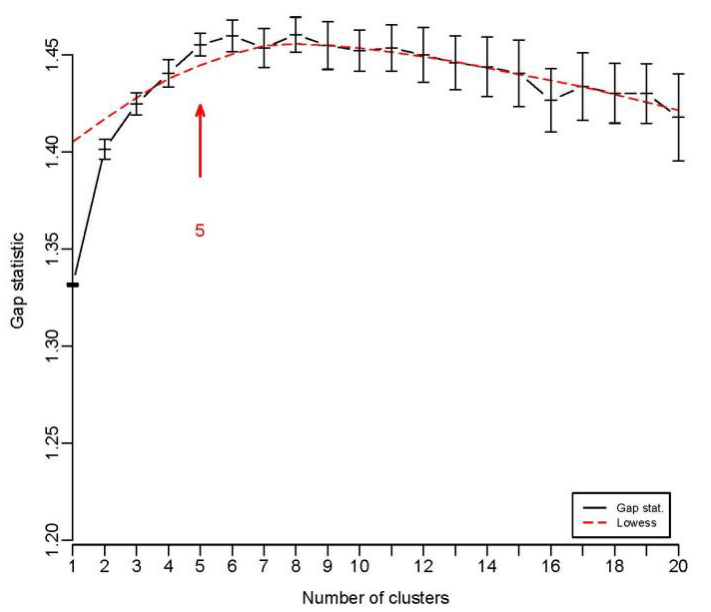
Probe sets.

We performed an agglomerative hierarchical cluster analysis on the Zcut values to determine how individuals cluster with respect to their expression profiles. The optimal number of clusters for the data set was estimated to be five based on the gap statistic measure (Fig. 1), and the clusters are hereafter referred to as *C1*, *C2*, *C3*, *C4*, and *C5*.

**Figure 2:**
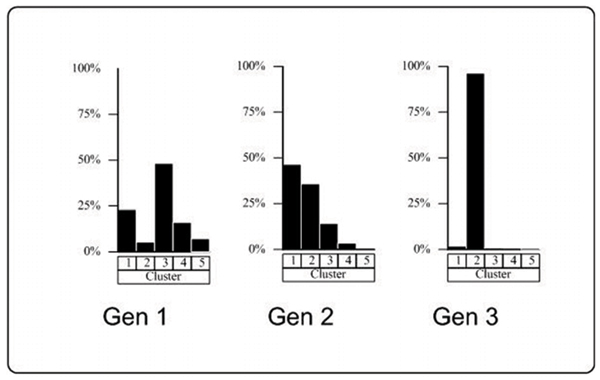
*p*-Value adjustments.

The clusters themselves appear to suggest a fairly strong age effect, as shown in Figure 2. The diagram shows the relation between cluster membership frequency and generational membership. On the basis of their gene expression variation alone, the grandparental generation (Gen 1) clusters primarily as *C1*, *C2*, and *C4*. The middle generation of parents (Gen 2) clusters as a mix of *C1*, *C2*, and *C3*, and the "sibship" generation (Gen 3) clusters almost exclusively together as *C2*. Because we are interested in finding evidence of heritable effects within the expression data, we sought to identify clusters that appear to represent mutually exclusive generations, which in this case would be *C2 *and *C4*.

**Figure 3:**
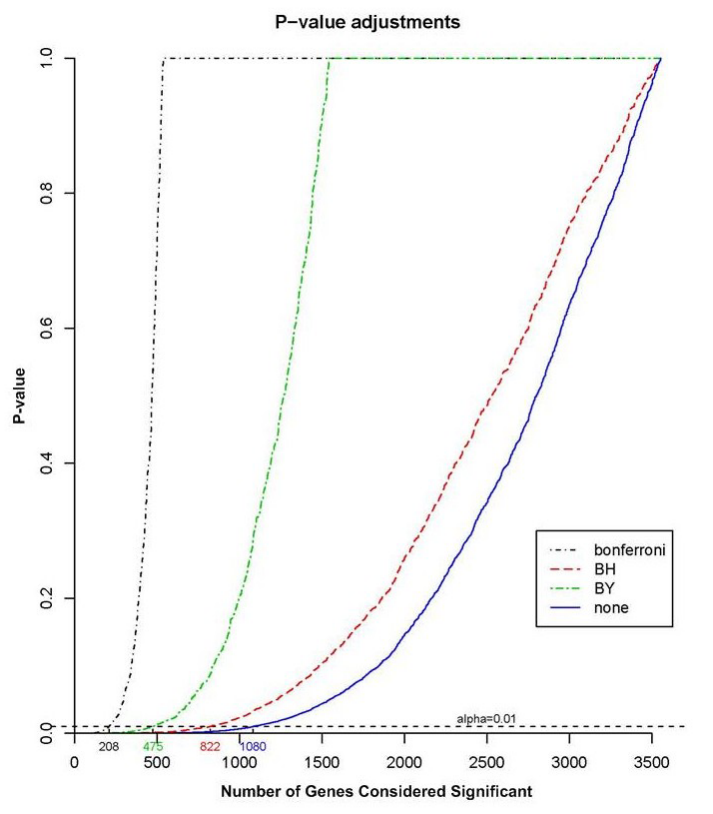
Gap statistic.

We also summarized the cluster analysis into a *heat map *designed to reveal specific points of aggregation within the data (Fig. 3), and our analysis of the heat map confirmed that *C2 *(shown in the leftmost block) and *C4 *(shown in the rightmost block) represent distinctive groups on the basis of their gene expression levels.

**Figure 4:**
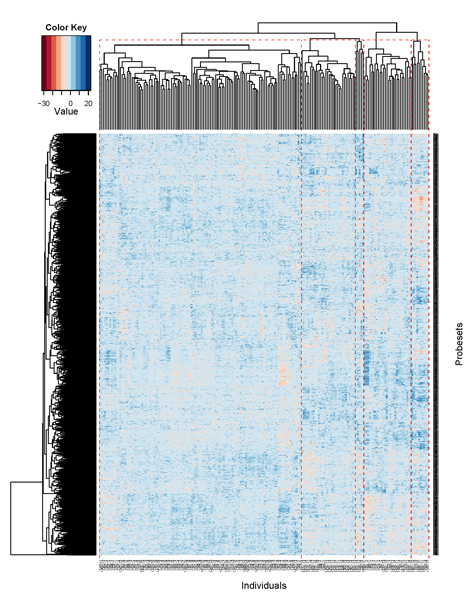
Generational composition by cluster.

After grouping individuals with respect to variation in their gene expression it is reasonable to ask which genes appear to be driving the inferred clustering. To answer this, we performed a one-way univariate ANOVA test for each gene to compare the between-group variation with the within-group variation of the calculated Zcut values. In each case, we calculated the *F *statistic and its *p*-value, and then applied a *p*-value correction for multiple testing to control the false discovery rate [12] setting the FDR at *α *= 0.01 (Fig. 4).

The one-way ANOVA test to identify genes that best explain the observed clustering revealed that 822 of the original 3554 genes were statistically significant for the clustering. This number reflects our attempt to control the false-discovery rate and appears to strike a reasonable balance between complete lack of correlation and the overly conservative correction obtained via the Bonferroni method (Fig. 3). The genes with the 10 largest *F *statistics were: *ADAR*, *MSH2*, *GLTSCR2*, *HNRPF*, *MIR16*, *PGLS*, *CYB5R3*, *HSPA8*, *PBK*, and *BAG2*. There is no overlap between the probe target locations for these genes.

Having characterized the data in terms of expression-driven clusters, we then turned to the question of how those clusters relate to the SNP genotypes. We recoded the SNP markers from the given coding: {0/0, 0/1, 1/1} to an additive coding: {0, 1, 2} such that the homozygote for the minor variant at a given genetic locus (i.e., the allele with the lowest frequency) is coded as 2. We then applied the multi-group ANOVA model described above to the SNP data, but instead of a no-baseline model, we compared the different clusters against each other, using the sibship cluster (*C2*) as the reference group.

Of the 2882 SNP loci analyzed under the model, 1480 had non-zero Zcut scores for at least one of the four possible clusters (*C1*, *C3*, *C4*, *C5*), and of this reduced set, 118 had nonzero Zcut scores for exactly one cluster. And of these, only 90 SNPs were applicable to the *C2 *vs. *C4 *comparison.

**Figure 5:**
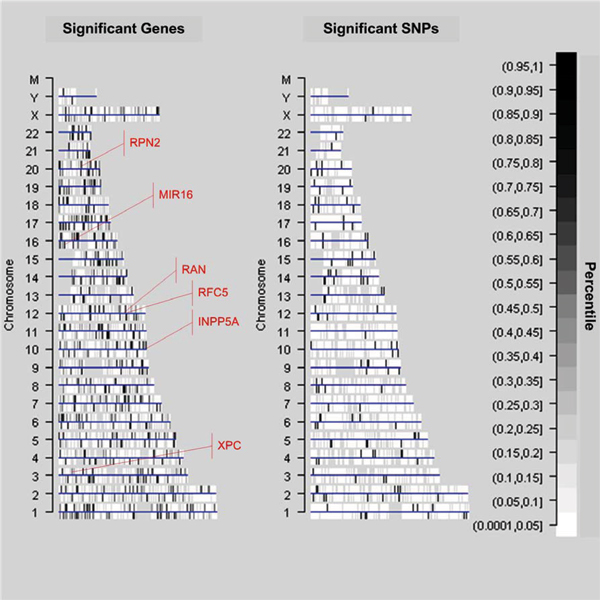
Comparative locations of significant genes and SNPs.

Our results are summarized by the diagram in Figure 5. Chromosomes are represented by straight lines parallel to the X-axis. Both significant genes and SNPs are represented by short perpendicular lines. The gene orientation is indicated by the position above (the '+' strand) or below (the '-' strand) the chromosome line. All gene locations for the Affymetrix HGFocus array are included in the plots. The white space represents locations for approximately 4500 unprobed genes.

## Conclusion

It seems likely that we have captured some type of age effect in the expression data as illustrated in Figure 2; however, because we have only limited information on what procedures were followed during data collection, we cannot rule out other possible explanations such as a batch effect.

We compared the chromosomal regions we found to be significant to those reported by other investigators [3,6], who, through linkage and hierarchical clustering analyses, found evidence for a regulation hotspot on 14q32. They found over 30 genes correlated with this region. We found 6 of those same genes significant in our analyses as well, including *RPN2*, *MIR16*, *RAN*, *RFC5*, *XPC*, and *INPP5A*. The second hotspot on 20q is the physical location of the gene *RPN2*. We found, as did Morley et al. [6], a physical relationship between these genes. *RAN *and *RFC5 *are located close together on 12q24 (Fig. 5).

While none of our significant SNPs correspond to reported associations with expression phenotypes, we did find two significant SNPs (rs945942 and rs945945) within a previously reported gene (*INPP5A*), both of which were found to significantly differentiate *C4 *from *C2*. Additionally, two of our most significant genes, *DDX17 *(chromosome 22), and *DSCR2 *(chromosome 21) have shown evidence of *cis*-acting and both *cis*- and *trans*-acting linkage, respectively [3,6].

Standard mapping techniques used in other studies (e.g., Morley et al. [6]) have been moderately successful in mapping *cis*- and *trans*-locations within the genome that cause variation in expression patterns in natural populations. Those methods typically analyze a single expression signal at a time, whereas we used a Bayesian ANOVA method to categorize groups of individuals by their similarity of gene expression profiles at a large number of genes. Such a method could, for example, be used for disease classification or heterogeneity classification when considering various phenotypes and other associated covariates. The second step of determining the extent to which SNPs discriminate between cluster memberships could be further used to differentiate between groups of individuals who have specific profiles. Another interesting possibility would be to identify group membership within an admixed population, based on the combination of expression phenotype and SNP genotype, in much the same manner as we have shown here. Because we use the gap statistic to determine the number of clusters, the method represents a completely data-driven approach to the problem of subdividing a given population with respect to genetic information.

We applied a whole-genome approach to determine the best set of SNPs that can explain cluster membership, and found that 90 SNPs best explained the difference between groups *C2 *and *C4*. Explicit modeling of the SNP/expression relationship that best explains this profile will enable us to model networks of genes that may be co-regulated. In most cases this type of clustering of phenotypic profiles (using expression or other phenotype data or some mixture thereof) can assist with incorporation of protective as well as susceptibility alleles inherent in the profiles.

In conclusion, our method can be generalized to address problems other than expression profile modeling. The key aspect we wish to emphasize is its applicability to variable selection when the number of variables is large compared to the number of observations. The ability to accurately partition a data set, based on the variance of SNP genotype or expression levels, into representative clusters of individuals is particularly appealing and likely to have many applications beyond those we have discussed here.

## Competing interests

The author(s) declare that they have no competing interests.
